# Supplementary figures and images for: Identification of TALE Transcription Factor Family and Expression Patterns Related to Fruit Chloroplast Development in Tomato (Solanum lycopersicum L.)
Source: Int J Mol Sci. 2022 Apr 19;23(9):4507. doi: 10.3390/ijms23094507 (PMC9104321; doi:10.3390/ijms23094507)

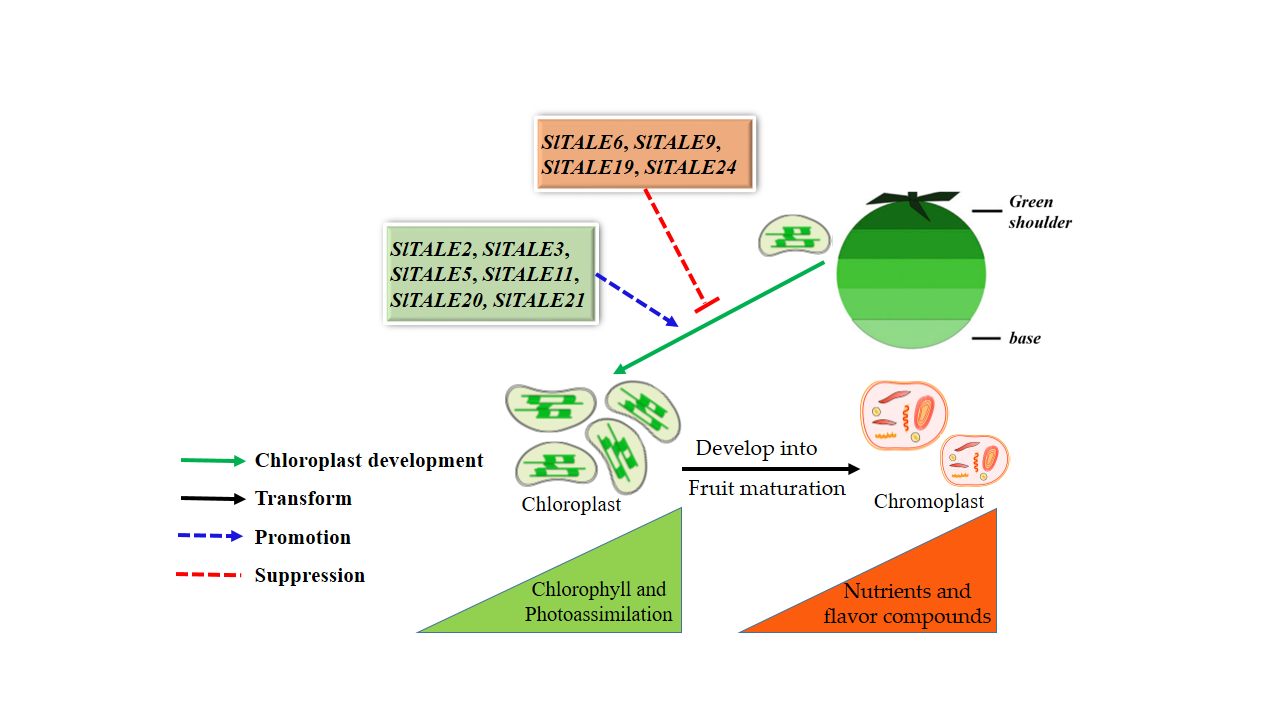

Supplement: Supplementary file 1 [file ijms-23-04507-s001.zip › Supplementary Files (Proofread)/illustrative figure.tif]
